# EGFR Mutant Structural Database: computationally predicted 3D structures and the corresponding binding free energies with gefitinib and erlotinib

**DOI:** 10.1186/s12859-015-0522-3

**Published:** 2015-03-14

**Authors:** Lichun Ma, Debby D Wang, Yiqing Huang, Hong Yan, Maria P Wong, Victor HF Lee

**Affiliations:** 1 Department of Electronic Engineering, City University of Hong Kong, Kowloon, Hong Kong; 2 School of Computer Science and Technology, Soochow University, Suzhou, China; 3 Li Ka Sing Faculty of Medicne, University of Hong Kong, Pokfulam, Hong Kong

**Keywords:** Epidermal growth factor receptor (EGFR), EGFR mutation database, Non-small-cell lung cancer (NSCLC), Tyrosine kinase inhibitor, Gefitinib, Erlotinib, Binding free energy

## Abstract

**Background:**

Epidermal growth factor receptor (EGFR) mutation-induced drug resistance has caused great difficulties in the treatment of non-small-cell lung cancer (NSCLC). However, structural information is available for just a few EGFR mutants. In this study, we created an EGFR Mutant Structural Database (freely available at http://bcc.ee.cityu.edu.hk/data/EGFR.html), including the 3D EGFR mutant structures and their corresponding binding free energies with two commonly used inhibitors (gefitinib and erlotinib).

**Results:**

We collected the information of 942 NSCLC patients belonging to 112 mutation types. These mutation types are divided into five groups (insertion, deletion, duplication, modification and substitution), and substitution accounts for 61.61% of the mutation types and 54.14% of all the patients. Among all the 942 patients, 388 cases experienced a mutation at residue site 858 with leucine replaced by arginine (L858R), making it the most common mutation type. Moreover, 36 (32.14%) mutation types occur at exon 19, and 419 (44.48%) patients carried a mutation at exon 21. In this study, we predicted the EGFR mutant structures using Rosetta with the collected mutation types. In addition, Amber was employed to refine the structures followed by calculating the binding free energies of mutant-drug complexes.

**Conclusions:**

The EGFR Mutant Structural Database provides resources of 3D structures and the binding affinity with inhibitors, which can be used by other researchers to study NSCLC further and by medical doctors as reference for NSCLC treatment.

## Background

As the primary type of lung cancer, non-small-cell lung cancer (NSCLC) has received growing attention from the researchers [1-3]. It is reported that about 85% of all the lung cancer patients are diagnosed as NSCLC [4]. One strategy commonly used in the treatment is to target the tyrosine kinase (TK) domain of epidermal growth factor receptor (EGFR) to interrupt the downstream signaling [5,6]. Reversible tyrosine kinase inhibitors (TKIs), such as gefitinib and erlotinib, are generally applied in this procedure. They are proven to be efficient for patients over a period of time, but a limited treatment outcome usually occurs because of mutation at EGFR TK domain [7,8]. According to statistics, about 10% to 15% of white patients and 30% East Asian patients experience a mutation of EGFR TK domain [4], and over one hundred mutation types have been found so far [9,10].

Structural information is available for just a few EGFR mutants from the Protein Data Bank (PDB) [11]. They are obtained with experimental methods, such as X-ray crystallography and nuclear magnetic resonance (NMR) spectroscopy [12]. These methods can produce high-resolution protein crystal structures, but they are usually very complex, costly and time consuming. Bioinformatics based methods have become very popular and successful in predicting protein structures [13,14]. Wang et al. [15] predicted EGFR mutant structures using the tools *scap* and *loopy*. Yarov‐Yarovoy et al. [16] employed Rosetta [17] to predict helical transmembrane protein structures. The binding free energy acts as a useful index to evaluate the binding affinity between mutants and drugs, and can be used as an important indicator of drug resistance. Zhou et al. [18] predicted EGFR mutation induced drug resistance based on the binding free energy, which was calculated with Amber [19]. As different mutations affect the EGFR structure and drug resistance level differently, a database of the EGFR mutant structures and the corresponding binding free energies with TKIs can provide a useful resource for further research and clinical guidance.

**Figure 1:**
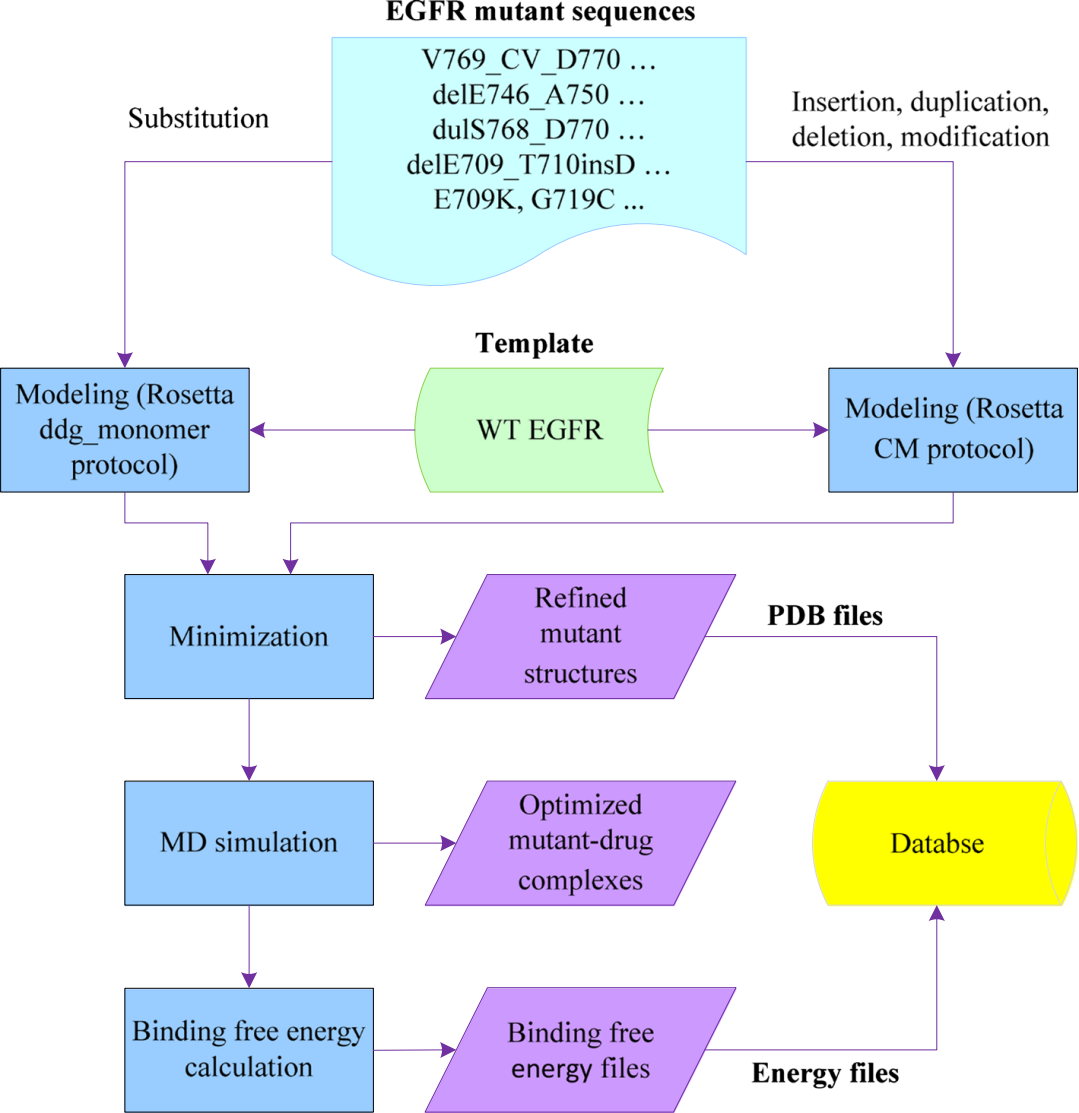
**Procedure used to build the EGFR Mutant Structural Database.** First, we applied Rosetta ddg_monomer protocol and comparative modeling (CM) protocol to predict EGFR mutant structures. Secondly, the predicted structures were refined with a minimization step using *sander* in Amber. Then a drug (gefitinib or erlotinib) was added to the mutant structures followed by MD simulation. Subsequently, we employed MM-GBSA in Amber to calculate the binding free energies of the EGFR mutants and the inhibitor. Finally, the refined mutant structures and their corresponding binding free energies with gefitinib and erlotinib were collected to establish the database.

In this study, we created an EGFR Mutant Structural Database, containing over one hundred EGFR mutants and their binding free energies with reversible TKIs gefitinib and erlotinib. We employed Rosetta [17] to generate the 3D structures of the EGFR mutants with the wild-type (WT) EGFR. Then Amber [19] was used to optimize the structures and compute the binding free energies with gefitinib and erlotinib. The procedure we have used to build the database is shown in Figure 1.

## Methods

### Data collection

The EGFR mutation types were obtained from the EGFR Mutation Database (http://www.cityofhope.org/egfr-mutation-database) [9] and the Queen Mary Hospital in Hong Kong [10]. The EGFR Mutation Database is a public database, while the data from Queen Mary Hospital in Hong Kong were obtained through several clinical projects and all of these projects had ethics approvals. None of any data entries contains patient identity. Before the commencement of this study, we obtained approval and permission from Institutional Review Board of the University of Hong Kong/Hospital Authority Hong Kong West Cluster to use the data from Queen Mary Hospital. The mutations locate at exons 18 to 21 of the EGFR TK domain. Specifically, there are 112 mutation types, including 95 from 774 NSCLC patients of the Mutation Database and 17 from 168 patients of the Queen Mary Hospital in Hong Kong. These mutation types are named according to their corresponding changes of the amino acid sequences relative to the WT sequence (Table 1). In the mutation representation, A and B, locating at positions p and q respectively, represent two residues in the protein sequence. I is a single residue or a residue list, and C and D are two other residues.

**Table 1:** **The naming rules of EGFR mutations**

**Name**	**Representation**	**Example**	**Description**
Insertion	Ap_I_Bq	V769_ASV_D770	Residues insertion
Deletion	delAp_Bq	delE746_S752	Residues deletion
Duplication	dulAp_Bq	dulA767_V769	Residues duplication
Modification	delAp_BqinsI	delE746_A750insAP	Combination of residues deletion and insertion
Substitution	ApB	T790M	Single-point mutation
	ApC_BqD	E709A_G719A	Double-point mutation

The crystal structures of EGFR mutants with L858R and G719S are available from PDB [11]. Other EGFR mutants, used for calculating binding free energies with gefitinib and erlotinib, were generated based on the template structures “2ITY” and “1 M17” respectively downloaded from PDB.

### Point mutation modeling

Residue substitution accounts for more than 60% (Table 2) of all the mutation types in our database. Single-point mutation is a replacement of an amino acid in the protein sequence with another, while double-point mutation occurs when the amino acids at two positions are replaced. In this paper, ddg_monomer in Rosetta was employed to generate the residue substitution mutants. This application takes the WT EGFR crystal structure and the mutant sequence as inputs, and the output is the structural model of the mutation with the side-chain replaced. Two main protocols are available in this procedure, the high-resolution protocol [20] and the low-resolution protocol [21]. The high-resolution protocol allows the backbone with a limited relaxation while the low-resolution protocol makes the backbone fixed. We adopted the high-resolution protocol to generate the EGFR single-point and double-point mutations. First, the side-chain at the mutant position is replaced and the Rosetta’s standard side-chain optimization module is applied to optimize the rotamers at all residues of the protein. Subsequently, gradient-based minimization is adopted to produce the minimized structures. As the high-resolution protocol allows the backbone a limited freedom, C_α_-C_α_ distance restraints are used in the optimization process in order to prevent the backbone moving too much from the start conformation.

**Table 2:** **Distribution of EGFR mutations**

**Name**	**Number of mutations**	**Number of patients**	**Percentage of all mutation types**	**Percentage of all the patients**
Insertion	7	9	6.25%	0.96%
Deletion	6	285	5.36%	30.25%
Duplication	6	18	5.36%	1.91%
Modification	24	120	21.42%	12.74%
Substitution	69	510	61.61%	54.14%

### Homology modeling

We employed the homology modeling (also known as comparative modeling (CM)) [22] protocol in Rosetta to generate the mutations of amino acids insertion, deletion, duplication and modification relative to WT EGFR. Homology modeling is widely used in predicting protein structures as it can often provide reliable and accurate structural models [23-25]. It provides a way to fill the large gap between the increasing number of available protein sequences and the protein crystal structures obtained from experimental methods [26].

Before model construction, several files (target and template protein sequences, template PDB file, mutant-aligned sequences, fragment library and secondary structure file of the target) should be prepared first. Selection of a template is very important because it can affect the accuracy of the predicted structure. In our studies, the crystal structures of EGFR TK domain “2ITY” and “1 M17” are selected as templates to generate the mutants. After the template is determined, mutant sequences are aligned to the template sequence with multiple-sequence alignment program ClustalW [27]. The fragment library includes short peptide backbone fragments, which can play an important role in the construction of variable regions. We employed the fragment picker protocol in Rosetta to pick fragments, which can help to establish models more efficiently and accurately by enabling rapid search of the conformational space. Moreover, PSIPRED [28] are used to obtain the target’s secondary structure file. After all these files prepared, the CM protocol [22] in Rosetta are applied to build the well-aligned regions and the missing parts are rebuilt using loop modeling with the fragment library. Finally, a full-atom refinement step is performed to the models and clustering method is used to select models.

### Model assessment

The models predicted with software simulation may not be accurate, thus the verification and assessment of the predicted models become very important. Two methods are often adopted to assess the predicted models with software simulation, computing the energy of the model and evaluating the similarity with a given characteristic between the predicted model and the real structure [26]. In this paper, we used physics-based energies of the predicted EGFR mutants to assess the accuracy of the 3D structures. The full atom energy scoring function was employed to calculate the energies of all the structures and the one with the minimum energy was identified as the finally predicted structure. Using the function, each predicted structure is scored with a series of parameters (Lennard-Jones interactions, solvation, residue pair interactions, van der Waals, hydrogen bonding, Ramachandran torsion preferences, rotamer self-energy and unfolded state reference energy) and their corresponding weights [29]. The total score of a predicted model is defined as the weighted sum of all the scoring parameters.

### Molecular dynamics (MD) simulation

After the predicted EGFR mutant structures were obtained, we optimized these structures using MD simulation in Amber [19]. The simulation is conducted in a solvent environment, thus, an octahedron water box (TIP3P model, 10.0-angstrom (Å)) is added to the structure with *tleap* in Amber. In order to describe the molecule interactions, the following molecular force field is adopted in Amber.

1 $$ \begin{array}{l}V(r)={E}_{bonded}+{E}_{nonbonded}\\ {}={\displaystyle \sum_{bonds}{K}_b{\left(b-{b}_0\right)}^2}+{\displaystyle \sum_{angles}{K}_{\theta }{\left(\theta -{\theta}_0\right)}^2}\\ {}+{\displaystyle \sum_{dihedrals}\left({V}_n/2\right)\left(1+ \cos \left[n\phi -\delta \right]\right)}\\ {}+{\displaystyle \sum_{nonbij}\left({A}_{ij}/{r}_{ij}^{12}\right)-\left({B}_{ij}/{r}_{ij}^6\right)+\left({q}_i{q}_j/{r}_{ij}\right)}\end{array} $$

The total energy is composed of bonded term *E*_*bonded*_ and non-bonded term *E*_*nonbonded*_. In Equation (1), the bonded energy which is related to the covalent bonds consists of bond stretching (where *K*_*b*_ is an empirical stretching force constant, *b* and *b*_*0*_ are the actual and empirical bond lengths respectively), angle bending (where *K*_*θ*_ is a constant, *θ* and *θ*_*0*_ are the actual and empirical bond angles respectively), and torsion terms (where *V*_*n*_ is the barrier to free rotation for the empirical bond, *n* is rotation periodicity, *ϕ* stands for torsion angle, and *δ* represents the angle when the potential reaches its minimum value). The non-bonded energy includes van der Waals (where *A*_*ij*_ and *B*_*ij*_ describe the depth and position for a pair of non-bonded interacting atoms respectively, and *r*_*ij*_ is the interatomic distance) and the long-range electrostatic terms (where *q*_*i*_ and *q*_*j*_ are point charges, and *r*_*ij*_ is the interatomic distance). In our simulation, we employed the ff99SB force field, which is a broad application of the basic force field. After solvating the complex and adding force filed, we conducted a minimization step to the entire system with *sander* in Amber. The result from the optimization process is our refined mutant structure.

With *MatchMaker* in UCSF Chimera [30], we aligned the optimized structure to the template complex “2ITY” (EGFR-gefitinib complex) or “1 M17” (EGFR-erlotinib complex) to obtain the mutant-drug complex. Then Amber was used to optimize these complexes. Similarly, the complex was solvated in a TIP3P water box (10.0 Å) and the ff99SB force filed was adopted. In order to conduct the production MD, we need to equilibrate the solvated complex using *sander* in Amber. First, 1000 circles of minimization were adopted to remove any bad contacts and make the structure relaxed. In this procedure, steepest descent algorithm was used for the first 500 steps and conjugate gradient algorithm was applied for the second 500 steps. Then 50 picosecond (ps) of heating and 50 ps of density equilibration were conducted to reach the temperature about 300 K and the density around 1 grams/ml. Subsequently, equilibration of constant pressure at 500 ps was carried out at the temperature of 300 K. All these simulations were conducted with shake on hydrogen atoms, and Langevin dynamics was used to control the temperature. Several parameters, such as temperature, density, total energy and root-mean-square deviation (RMSD) were finally used to verify that the equilibration of the system. When the system is equilibrated, we proceeded to run the production MD for a total of 2 ns and recorded the coordinates every 10 ps.

### Binding free energy calculation

The binding free energy of each mutant-drug complex is calculated based on the motion trajectories, which are generated during the production MD simulation. The MM-GBSA method in Amber tools was applied to calculate the binding free energies of EGFR mutants and reversible TKIs (gefitinib and erlotinib). The aim of this procedure is to obtain the free energy difference between the bound and unbound state of two solvated molecules. However, in a solvent environment, the solvent-solvent interactions account for most energy contributions and the fluctuations of the total energy would be an order of magnitude greater than the binding energy. Therefore, the binding free energy is calculated as follows by means of thermodynamic cycle in solvent and vacuum environment.

2 $$ \begin{array}{l}\varDelta {G}_{bind, solv}=\varDelta {G}_{bind, vacuum}+\varDelta {G}_{solv, complex}\\ {}-\left(\varDelta {G}_{solv, ligand}+\varDelta {G}_{solv, receptor}\right)\end{array} $$

where ∆*G*_*bind,solv*_ and ∆*G*_*bind,vacuum*_ represent the free energy difference of bound and unbound state of a complex in solvent and vacuum environment respectively, and ∆*G*_*solv,receptor*_, ∆*G*_*solv,ligand*_ and ∆*G*_*solv,complex*_ stand for the changes of free energies of the receptor, ligand and complex between solvent and vacuum environment, respectively.

We calculated the binding free energies of EGFR mutants with gefitinib and erlotinib. MM-GBSA in Amber derives the interaction energy and solvation free energy for the receptor, ligand and complex respectively. The energy of each molecular is composed of several terms, including van der Waals force (VDWAALS), electrostatic energy (EEL), the electrostatic contribution to the solvation free energy (EGB) and nonpolar contribution to the solvation free energy (ESURF). The total binding free energy is given by ∆G along with error values.

## Results and discussion

### Data analysis

According to the naming rules, 112 EGFR mutation types of the 942 NSCLC patients are divided into five groups, including insertion, deletion, duplication, modification and substitution. We counted the number of mutation types as well as the corresponding patients of each mutation type (Table 2). From Table 2, substitution accounts for more than half of EGFR mutation types and the number of patients. Although deletion just takes up 5.36% of all the mutation types, 285 cases belong to this group and they hold 30.25% of all the patients.

Among all the 112 mutation types, several of them take up the majority of the patients, such as L858R, delE746_A750 and delL747_P753insS. We listed the top 10 common mutations among the 942 patients in Table 3. Top two of them (L858R and delE746_A750) accounts for more than half of all the patients in total. Specifically, 388 cases experienced a mutation of L858R at exon 21, taking up 41.19%, and 264 patients suffered from delE746_A750 (deletion of amino acids at exon 19), which accounts for 28.03%.

**Table 3:** **Most common EGFR mutation types**

**Mutation types**	**Number of patients**	**Percentage**	**Position (Exon)**
L858R	388	41.19%	21
delE746_A750	264	28.03%	19
delL747_P753insS	43	4.56%	19
delE746_S752insV	16	1.70%	19
G719S	10	1.06%	18
delL747_T751	10	1.06%	19
delL747_T751insP	10	1.06%	19
L861Q	10	1.06%	21
G719C	9	0.96%	18
delE746_T751insA	9	0.96%	19

Moreover, we analyzed the mutations and the number of patients at each exon (Table 4). From Table 4, the number of occurrences of most mutation types at each exon is less than or equal to 3. For example, there are 14 mutation types at exon 18 with the number of occurrences less than or equal to 3 and only 3 mutations with occurrences more than 3. However, those mutations with the number of occurrences more than 3 often occupy more patients than all other mutations with the number of occurrences less than or equal to 3. For instance, 371 patients have 10 mutation types while just 38 patients have 26 mutation types at exon 19. In addition, exon 19 (32.14%) occupies the most mutation types, and exon 21 (44.48%) accounts for the greatest number of the NSCLC patients.

**Table 4:** **Distribution of mutation types and the number of patients by mutation position**

**Mutation position**	**Number of occurrences**	**Number of mutation types**	**Number of patients**	**Percentage of all mutation types**		**Percentage of all the patients**	
Exon 18	≤3	14	19	12.5%	15.18%	2.02%	4.89%
	>3	3	27	2.68%		2.87%	
Exon 19	≤3	26	38	23.21%	32.14%	4.03%	43.41%
	>3	10	371	8.93%		39.38%	
Exon 20	≤3	25	34	22.32%	25.89%	3.61%	6.05%
	>3	4	23	3.57%		2.44%	
Exon 21	≤3	18	21	16.07%	17.86%	2.23%	44.48%
	>3	2	398	1.79%		42.25%	
Others	≤3	10	11	8.93%	8.93%	1.17%	1.17%

### EGFR mutant structure prediction

**Figure 2:**
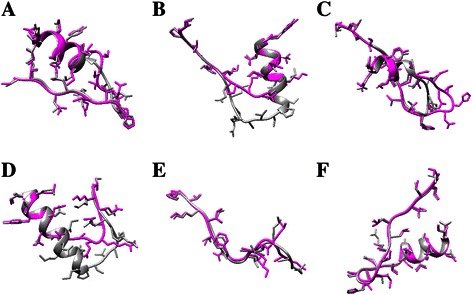
**Comparison between local changes of the predicted structures and WT EGFR. (A** **-F)** display an example of insertion, deletion, duplication, modification and substitution (single-point and double-point), corresponding to V769_CV_D770, delE746_S752, dulN771_H773, delT751_I759insN, L165Q and S72IV78M, respectively. The dark gray chains represent the WT structures while the magenta chains stand for the predicted ones with Rosetta.

We employed Rosetta to generate the EGFR mutants based on the template structures “2ITY” and “1 M17”. As the crystal structures of L858R and G719S are available from PDB, we took the two models as the 3D mutant structures. The procedure of predicting EGFR mutants using Rosetta has been discussed in *Point Mutation Modeling* and *Homology Modeling* parts of the Materials and methods Section. After the mutant structures were obtained, we employed *sander* in Amber to conduct a short 1000 steps of minimization to remove any bad contacts and find out the nearest local minima. Then the refined structures were saved as our predicted EGFR mutants for further analysis. Figure 2 shows the mutation neighborhood of our predicted structures and the WT structure. We employed UCSF Chimera [30] to display these structures.

**Figure 3:**
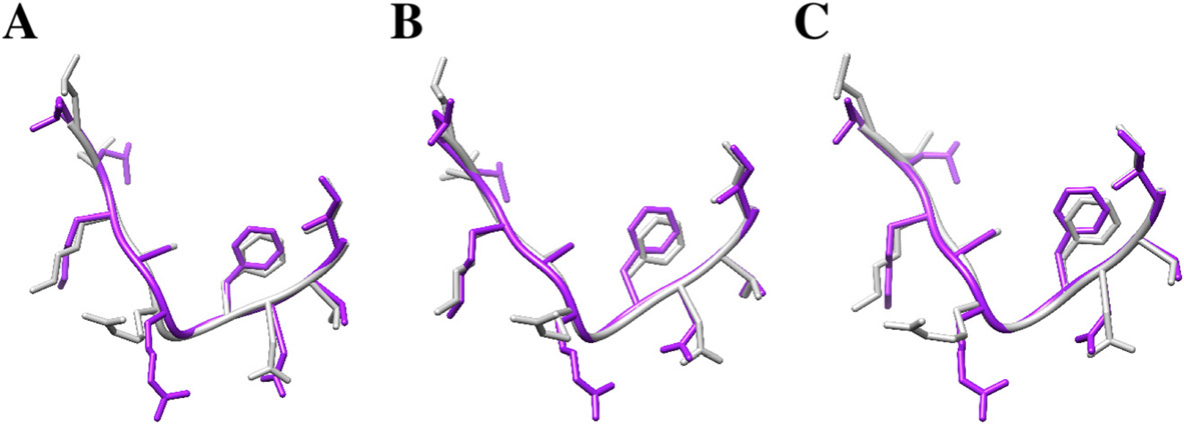
**Comparison of the predicted structures and the actual structure of L858R. (A-C)** show local comparison of predicted structureE01, structureE02, structureE03 and the actual structure “2ITZ”, respectively. The dark gray chains represent the actual structures while the purple chains stand for the predicted ones using Rosetta.

In the EGFR Mutant Structural Database, computationally predicted structures are provided. These structures were selected according to the full atom energy scoring function. To examine the accuracy of the predicted structures, we made a comparison of the predicted structures and the actual structure of L858R, the most common mutation of the EGFR TK domain. Ten amino acids close to the mutation position were selected in order to see the local differences. Using UCSF Chimera, we aligned the predicted structures (structureE01, structureE02 and structureE03 from http://bcc.ee.cityu.edu.hk/data/EGFR.html) to the actual structure “2ITZ” from PDB. Figure 3 shows the comparison of the actual structure and the structures generated based on Rosetta. The backbones of the predicted structures are consistent with the actual one. In addition, the backbone RMSDs of the three pairs of structures are 0.725 Å, 0.562 Å and 0.559 Å respectively, which confirms the good accuracy of our prediction procedure.

**Figure 4:**
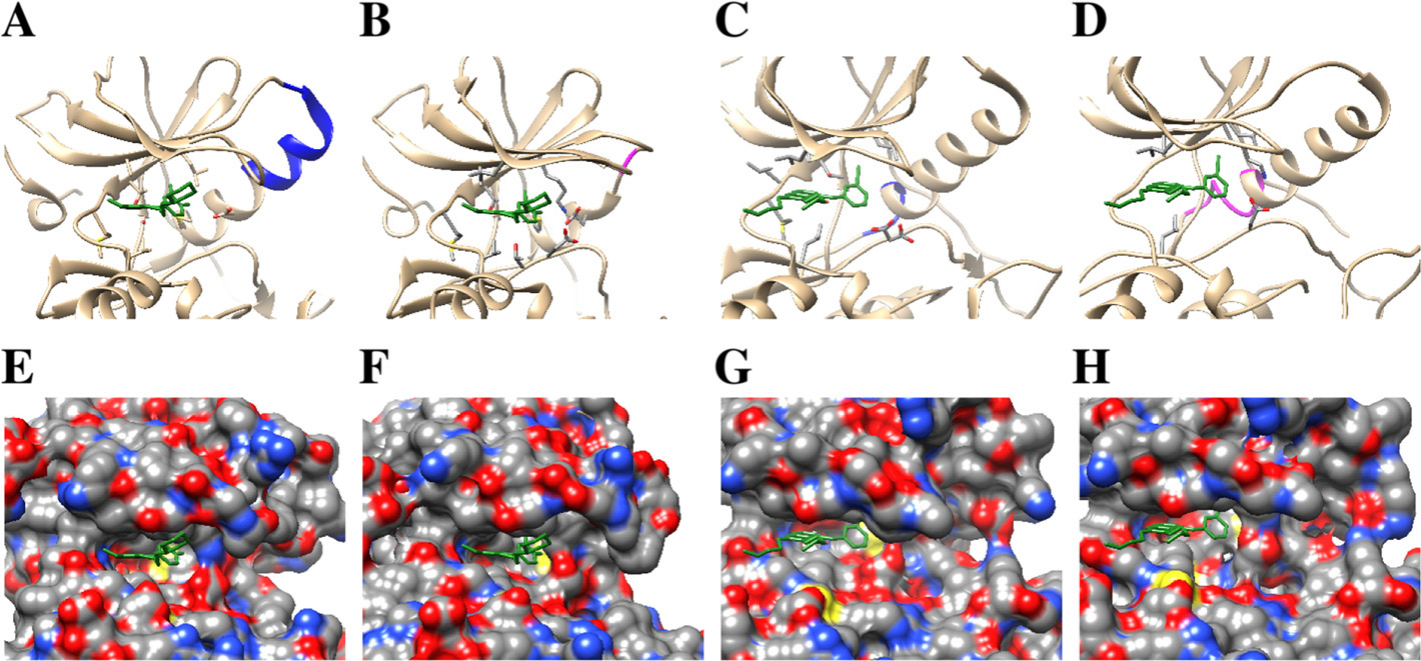
**Comparison of the WT EGFR-drug complex and mutant-drug complex structures. (A)** and **(B)** display the WT EGFR and the mutant structure delT751_I759insS with gefitinib. **(C)** and **(D)** show the WT structure and the mutant dulA767_V769 with erlotinib. **(E**-**H)** present the corresponding solvent-excluded molecular surfaces of **(A)** to **(D)**, and the drug binding pockets can be seen very clearly. In **(A)** to **(D)**, the mutant sites are shown in magenta while the original sites are presented in blue. In addition, drugs are colored green.

Once the optimized mutant structures were obtained, we aligned these structures to the WT EGFR using *MatchMaker* in UCSF Chimera. Inhibitors were added to the structures followed by several optimization steps to these mutant-drug complexes using MD simulation. Before MD simulation, the mutant-drug complex should first be solvated in a TIP3P water box (10.0 Å) and the ff99SB force filed was needed in order to describe the molecule interactions. Then a series of refinement operations (minimization, heating, density equilibration and constant pressure equilibration) were conducted with *sander* in Amber as introduced in the *Molecular dynamics (MD) simulation* part of the Materials and methods Section. Figure 4 shows the comparison of minimized EGFR mutant-drug complex and WT EGFR-drug complex structures.

### Binding free energy calculation

**Figure 5:**
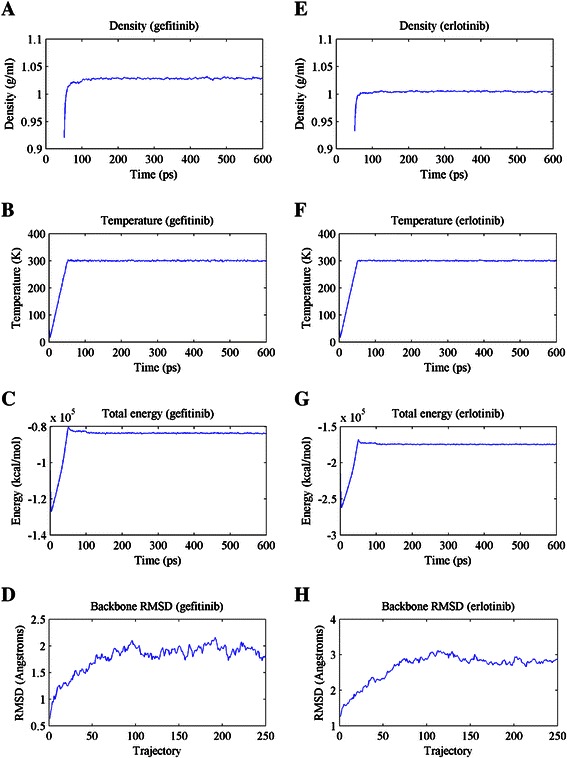
**The density, temperature, total energy and RMSD of delE746_A750 with gefitinib (A**-**D) and erlotinib (E**-**H) during the equilibration period.**

Binding free energies are calculated based on the trajectories determined during the production MD simulation process. Before running the production MD simulation, we should make sure that the solvated complex has equilibrated. For this, the terms of temperature, density and total energy of the system are examined. Moreover, the protein backbone RMSD is checked in order to see whether the conformational stability has been achieved. Figure 5 shows the verification terms of the mutant delE746_A750 with gefitinib and erlotinib during the equilibration period. From Figure 5, the computational processes for the density, temperature and total energy of delE746_A750 with gefitinib and erlotinib are all converged at last, which can be used as evidence for system equilibration. The backbone RMSD is in an acceptable level although it is not converged completely for each system.

After the system reaches equilibration, production MD simulation is conducted. Similarly, we still check equilibrium phase space of the system by the density, temperature, total energy and backbone RMSD during the production phase in order to obtain good simulation results. Then, with the trajectories produced in the production MD simulation process, binding free energies are calculated using MM-GBSA in Amber tools. The total binding free energies of WT EGFR and several common mutation types with gefitinib and erlotinib are shown in Table 5. Moreover, the standard deviations (SD) and standard error of the mean (SED) are also listed. If the binding free energy of the mutant and drug is low, generally, they are considered binding well with each other, which means the drug can inhibit the activation of EGFR mutant.

**Table 5:** **Binding free energies of WT EGFR-drug complex and several common mutation-drug complexes**

**EGFR**	**Binding free energy with gefitinib (kcal/mol)**			**Binding free energy with erlotinib (kcal/mol)**		
	**Total**	**SD**	**SEM**	**Total**	**SD**	**SEM**
WT	−43.8839	2.7576	0.1950	−41.2009	3.0544	0.2160
L858R	−46.0101	2.7728	0.1961	−45.1344	2.6856	0.1899
delE746_A750	−35.2995	2.9642	0.2096	−44.6007	3.3339	0.2357
delL747_P753insS	−28.5837	3.6056	0.2550	−38.1157	3.0238	0.2138
delE746_S752insV	−42.7755	3.1422	0.2222	−33.2645	2.9925	0.2116
G719S	−35.4427	2.7781	0.1964	−43.4964	3.3155	0.2344

**Figure 6:**
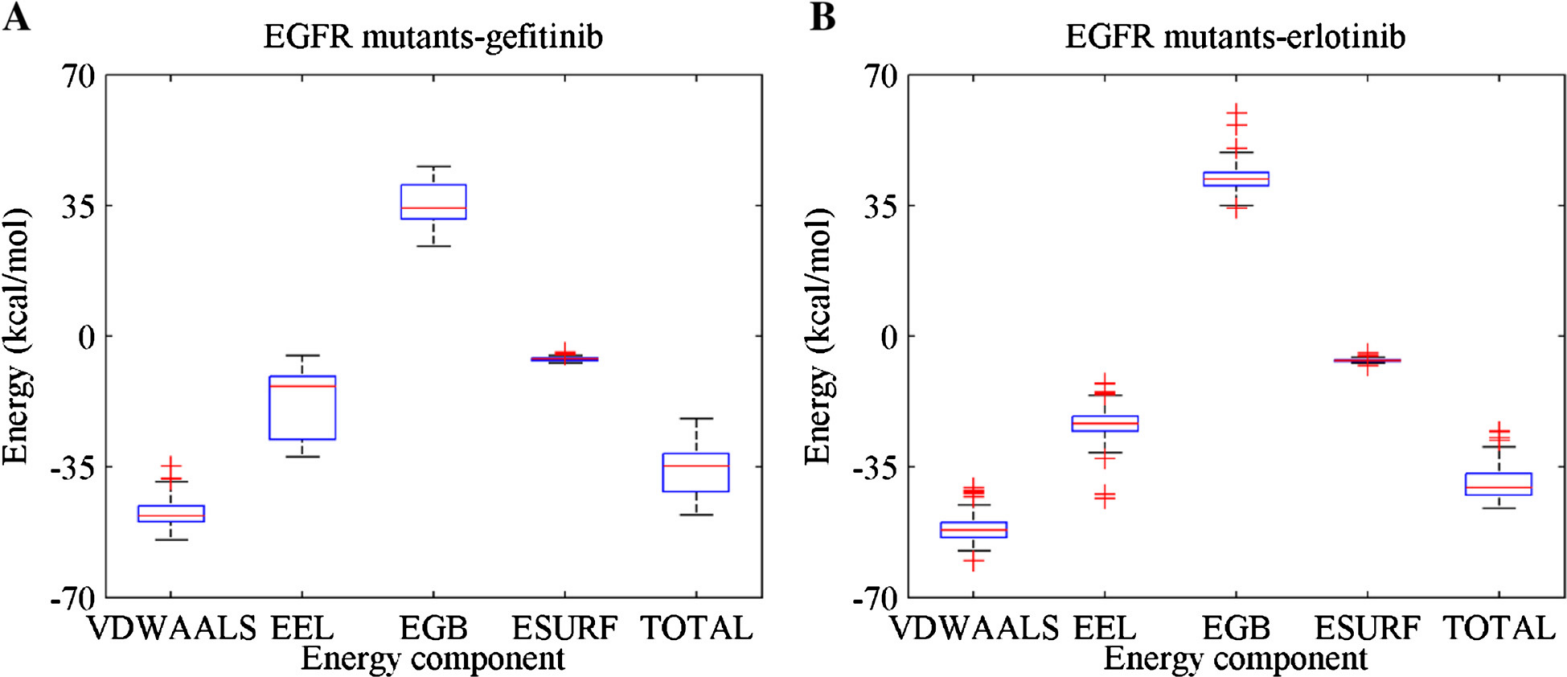
**Distribution of binding free energies of all the EGFR mutants with (A) gefitinib and (B) erlotinib.** The energy components and the total energies are shown in the diagrams.

The total binding free energy is composed of several energy components, including VDWAALS, EEL, EGB and ESURF. Figure 6 shows the distribution of each energy component and the total binding free energies of all the EGFR mutants and the inhibitors. As shown in Figure 6A and B, the total energy and energy components (VDWAALS, EEL and EGB) of the EGFR mutant-gefitinib complexes are distributed in a wider range than those of EGFR mutant-erlotinib complexes.

### Comparison of the EGFR mutant structural database and other EGFR-related databases

Several EGFR-related databases are available publicly, such as the EGFR Mutation Database (http://www.cityofhope.org/egfr-mutation-database) [9], the Catalogue of Somatic Mutations in Cancer (http://cancer.sanger.ac.uk/cancergenome/projects/cosmic/) [31], EGFR Inhibitor Database (http://crdd.osdd.net/raghava/egfrindb/) [32] and the widely used PDB [11]. The EGFR Mutation Database contains the mutant position information as well as the response to inhibitors of the NSCLC patients. The COSMIC stores somatic mutation data of human cancer. These databases just provide the sequence information of the mutations. The EGFR Inhibitor Database contains biological and chemical information of the EGFR inhibitors. PDB provides crystal structures of proteins, nucleic acids, and complex assemblies obtained from experimental methods, such as X-ray or NMR. However, only a few EGFR mutant structures are available because of the high cost of experiments. The EGFR Mutant Structural Database presented in this paper contains 3D structures of 112 kinds of EGFR mutants. Moreover, the binding free energies of the mutant and inhibitors are provided to show the binding affinity. The structural information is very helpful to conduct protein docking, hydrogen bond analysis, and protein-drug complex simulation, which are very important in the studying of drug resistance mechanisms.

In our previous work [15,33], the molecular mechanisms have been identified from the aspects of geometric properties of mutant structures and the binding free energies with gefitinib and erlotinib. In [33], with 30 mutant structures generated by Rosetta, we analyzed local surface changes of the binding pocket relative to the wild-type EGFR using alpha shape modeling. Moreover, we conducted a correlation analysis about the geometric properties and the pre-recorded progression-free survival (PFS) in the treatments. Results show that the curvature of the binding pocket surface plays an important role in the prediction of EGFR mutation-induced drug resistance. In [15], we identified drug resistance mechanisms from the binding free energies with inhibitors (gefitinib and erlotinib) as well as some personal features of 168 patients (belonging to 37 mutation types). Extreme learning machine method was employed to build a classification model and resistant subjects were successfully identified. Overall, the molecular mechanisms of drug resistance are closely related to the mutant structures and the binding affinity with inhibitors. Thus, the EGFR Mutant Structural Database we built here is very useful to other researchers and medical doctors for further studying or clinical guidance.

## Conclusions

In this work, we created an EGFR Mutant Structural Database, composed of computationally predicted 3D structures of the EGFR mutants and the corresponding binding free energies with gefitinib and erlotinib. In our database, 112 kinds of mutants were collected from 942 NSCLC patients. We categorized the mutants into five groups (insertion, deletion, duplication, modification and substitution), and substitution accounts for 61.61% of the EGFR mutation types and 54.14% of all the patients. As the most common mutation type, L858R covers 388 or 41.19% of all the patients. In addition, we analyzed the mutations at each exon. It shows that exon 19 (32.14%) possesses the most mutation types and exon 21 (44.48%) occupies the largest number of patients. With the mutant protein sequences and WT EGFR crystal structure, we predicted the EGFR mutation structures with Rosetta and optimized the structures using Amber. Finally, we calculated the binding free energies of EGFR mutants and the inhibitors (gefitinib and erlotinib). Our work provides a database of the EGFR mutant structures and their corresponding binding free energy with inhibitors. These resources can be used for further researches and clinical guidance, such as analyzing drug resistance of the EGFR mutants, which is a major problem during the treatment of NSCLC patients. The database is freely available at http://bcc.ee.cityu.edu.hk/data/EGFR.html.
